# Over-Expression of an R2R3 MYB Gene, *MdMYB108L*, Enhances Tolerance to Salt Stress in Transgenic Plants

**DOI:** 10.3390/ijms23169428

**Published:** 2022-08-21

**Authors:** Bingyang Du, Heng Liu, Kuntian Dong, Yong Wang, Yuanhu Zhang

**Affiliations:** 1 College of Life Science, State Key Laboratory of Crop Biology, Shandong Agricultural University, Tai’an 271018, China; 2 State Key Laboratories of Agrobiotechnology, Department of Pomology, College of Horticulture, China Agricultural University, Beijing 100193, China; 3 College of Life Sciences, Linyi University, Linyi 276005, China; 4 College of Life Sciences, South China Agricultural University, Guangzhou 510642, China

**Keywords:** apple, transcription factor, MdMYB108L, salt stress

## Abstract

Plants are affected by various abiotic stresses during their growth and development. In plants, MYB transcription factors are involved in various physiological and biochemical processes, including biotic and abiotic stress responses. In this study, we functionally analyzed MdMYB108L. We examined the transcriptional activity of MdMYB108L under salt stress and determined that the N-terminal domain of MdMYB108L, which was significantly induced under salt stress, has transcriptional activity. MdMYB108L overexpression increased the germination rate, main root length, and the antioxidant activity of catalase and peroxidase in transgenic *Arabidopsis* *thaliana* seeds, while reducing reactive oxygen species (ROS) accumulation. MdMYB108L overexpression also increased the photosynthetic capacity of hairy root tissue (leaves) under salt stress. In addition, the MdMYB108L transcription factor bound to the *MdNHX1* promoter positively regulated the transcription of the salt tolerance gene *MdNHX1* in apples, improving the salt stress tolerance of transgenic plants. These findings have implications for improving the agricultural yields of apple trees under salt stress.

## 1. Introduction

Salt stress is a critical factor limiting crop yields. Sodium is the main ionic component in salt stress environments [1]. The increase in sodium ions reduces the soil particle porosity, ultimately limiting the absorption of water, oxygen, and nutrients by plants. Under salt stress, plant cells transport and store excess sodium accumulated in the cytoplasm to the vacuoles. The increase in sodium ions can also affect various physiological and biochemical parameters in plant cells by dysregulating the osmotic potential, weakening photosynthesis, increasing ROS concentrations, and promoting metabolic disorders, adversely affecting the normal growth and development of plants [2].

Salt stress can significantly affect the abilities of plants to absorb water by affecting their osmotic potential. However, plants can respond to salt stress by stabilizing the cellular osmotic potential by synthesizing osmotic-adjustment substances [3]. To cope with excessive ROS produced under salt stress, plants develop various enzymatic and non-enzymatic defense mechanisms to curtail the damaging effects of ROS [4,5]. Antioxidant enzymes, such as glutathione peroxidase, ascorbate peroxide (APX), catalase (CAT), superoxide dismutase (SOD), and glutathione reductase, play an important role in the oxidative stress response [6,7]. Salt stress can trigger the closure of the stomata of plants, reducing their photosynthetic capacities. Besides, the inhibition of the dark-reaction process can further reduce CO_2_ fixation [8,9,10,11]. Stress responses in chloroplasts may induce photosystem photoinhibition, yielding excessive ROS [12,13,14,15].

MYB transcription factors are of great significance in regulating abiotic stresses in plants. The R2R3-MYB family of transcription factors plays a central regulatory role in various biological processes related to growth and development, and abiotic stress responses [16]. LcMYB1 confers salt tolerance to transgenic Arabidopsis [17]. StMYB1R-1 overexpression in potato plants improves the tolerance of transgenic plants to drought stress [18]. Heterologous expression of the soybean GmMYBJ1 gene enhanced the tolerance of transgenic Arabidopsis plants to drought and cold stress [19]. GbMYB5 also confers drought tolerance to cotton and genetically modified tobacco plants [20]. PcWRKY11, from *Polygonum cuspidatum*, enhances salt tolerance in transgenic *Arabidopsis thaliana* [21]. Overexpression of the apple MdMYB121 gene significantly enhanced the tolerance of transgenic tomato and apple plants to high salt and drought stress [22]. Overexpression of apple MdSIMYB1 in transgenic tobacco and transgenic apple lines enhanced transgenic plant tolerance to high salt, drought, and cold stress by upregulating stress-responsive genes [23]. *Scutellaria baicalensis* SbMYB8 overexpression in transgenic tobacco altered the expression levels of specific flavonoid biosynthesis-related genes, improving drought stress tolerance in transgenic plants [24]. Several members of the R2R3-MYB protein family are involved in AbA-dependent signaling pathways to regulate stress adaptation. In plants, AbA plays a central role in regulating the response to drought stress through the integrated cooperation of complex gene regulatory networks, promoting plant self-adaptation under water-deficient conditions [25]. MYB96 enhances plant resistance to drought stress by reducing stomatal opening and regulates later steps of lateral-root-developmental inhibition under drought stress conditions, a typical adaptive response in plants [26].

In model plants, HKT1 (high-affinity K^+^ transporter), SOS1 (salt overly sensitive 1), and NHX (Na^+^/H^+^ exchanger) were identified as key determinants of cellular Na^+^ homeostasis. While HKT1 and SOS1 control the net flux to the plasma membrane, NHX controls the transport of Na^+^ across the vacuolar membrane into the vacuole [27,28,29]. It is well known that NHX1 plays an important role in plant responses to salt stress. Overexpression of Arabidopsis NHX1 (AtNHX1) improves salt tolerance in many plant species, including *Arabidopsis thaliana* [30], tomato [31], wheat [32], soybean [33], and peanuts [34]. In contrast, T-DNA insertion mutants of AtNHX1 showed greater sensitivity to NaCl in Arabidopsis seedlings [35]. Furthermore, NHX1 reportedly plays an important role in cellular K^+^/Na^+^ homeostasis [36], tonoplast pH calibration [37], regulation of plant growth, flower development, and reproduction [38]. NHX1 overexpression seemingly improves salt tolerance in different plant species. For instance, AtNHX1 overexpression in Arabidopsis and AgNHX1 from the halophyte *Atriplex gmelini* in rice significantly increased the salt tolerance of transgenic plants [30,39,40]. Likewise, DmNHX1 overexpression in *Dendranthema morifolium* enhanced salt tolerance in transgenic *Arabidopsis thaliana* [41].

*Malus domestica* Borkh apples represent an economically important fruit crop, widely cultivated worldwide. Due to the large apple cultivation area, apples must cope with different planting environments, facing varying degrees of abiotic stress during the cultivation process, especially salt stress, which severely affects fruit yield and quality. In this study, we cloned and identified the salt-tolerant gene, *MdMYB108L*. We found that salt stress significantly induced *MdMYB108L* expression. *MdMYB108L* overexpression in *Arabidopsis* and apple hairy root tissue significantly enhanced the salt tolerance of transgenic plants. Further analysis showed that MdMYB108L positively regulated the transcription of the salt tolerance gene *MdNHX1* in apples, improving the salt tolerance of transgenic plants. This study explored the physiological basis and molecular mechanism of MdMYB108L under salt stress. In addition, it has important reference significance for understanding the MYB of other species involved in abiotic stress.

## 2. Results

### 2.1. Amino Acid Sequence Analysis and Transcription Activation Activity Analysis of MdMYB108L

**Figure 1:**
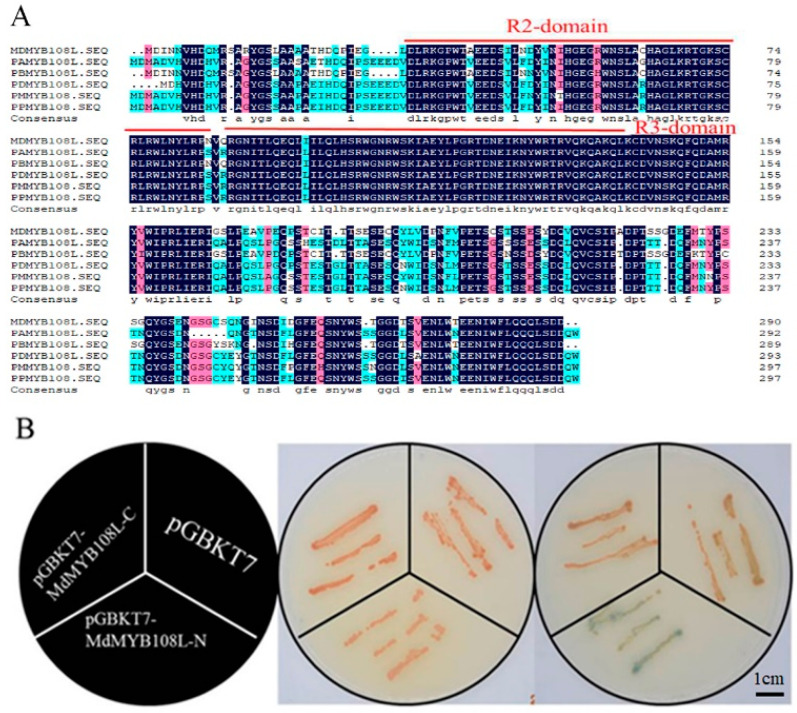
Amino acid-sequence analysis and transcription activation activity analysis of MdMYB108L. (**A**) Amino acid-sequence alignment of MdMYB108L (GenBank accession numbers XM_021957017.1, XM_009368914.2, XM_034352828.1, XM_008229595.1, and XM_007221459.2), usingDNAMAN 9 software (Lynnon LLC, San Ramon, CA, USA) with default parameters. (**B**) Transcriptional activation analysis of *MdMYB108L* in yeast cells. Bar = 1 cm.

The amino acid sequences of MYB108L proteins from different species were downloaded from the NCBI website, and the amino acid sequences were compared using DNAMAN software. We found that MdMYB108L (GenBank accession number: MDP0000823458) had a conserved R2R3 domain (Figure 1A). This finding shows that MdMYB108L belongs to the R2R3-MYB family and that it may have similar functions to other members of the R2R3-MYB family and play a role in plant stress.

We divided the MdMYB108L protein into two segments: the sequences containing the N-terminus (amino acids 1–145) and the C terminus (amino acids 146–290). The experimental results showed that, compared with control transformants, yeast transformed with the C-terminal target fragment did not show changes on the chromogenic medium. In contrast, yeast transformed with the N-terminal target fragment induced α-galactosidase activity in the medium and, thus, appeared blue on the chromogenic medium (Figure 1B). These findings show that the MdMYB108L protein has transcriptional activation activity and that the transcriptional activation region resides in the N-terminus.

### 2.2. Identification of MdMYB108L as a Salt-Responsive Transcription Factor

**Figure 2:**
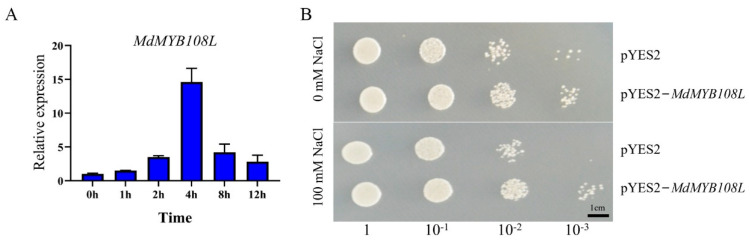
Identification of the *MdMYB108L* gene in response to salt stress. (**A**) qRT-PCR analysis of *MdMYB108L* expression in wild −type apple seedlings under salt stress. ‘Gala’ apple seedlings were treated with 200 mM NaCl for the indicated times (0, 1, 2, 4, 8, or 12 h). The expression level observed at 0 h was set to 1. (**B**) Effects of *MdMYB108L* on the growth properties of yeast strains under salt stress. Yeast cells expressing *MdMYB108L* (pYES2−*MdMYB108L* vector transformants) or not (empty pYES2 vector transformants) were grown on medium containing 0 or 100 mM NaCl for 3 days. Bar = 1 cm.

We treated apple leaves with 200 mM NaCl and performed qRT-PCR for relative quantitative analysis of gene expression. The quantitative results showed that under salt stress, *MdMYB108L* expression tended to initially increase then decrease, especially after 4 h. *MdMYB108L* gene expression was approximately 14 times higher than that of the control group (Figure 2A); thus, salt stress significantly induced *MdMYB108L* expression. Yeast cells harboring the pYES2-*MdMYB108L* grew better under salt stress compared with the control group (Figure 2B). These results indicate that *MdMYB108L* overexpression increases the tolerance of yeast cells to salt stress.

### 2.3. MdMYB108L Overexpression in Arabidopsis Decreased the Sensitivity to Salinity Stress at the Germination Stage

**Figure 3:**
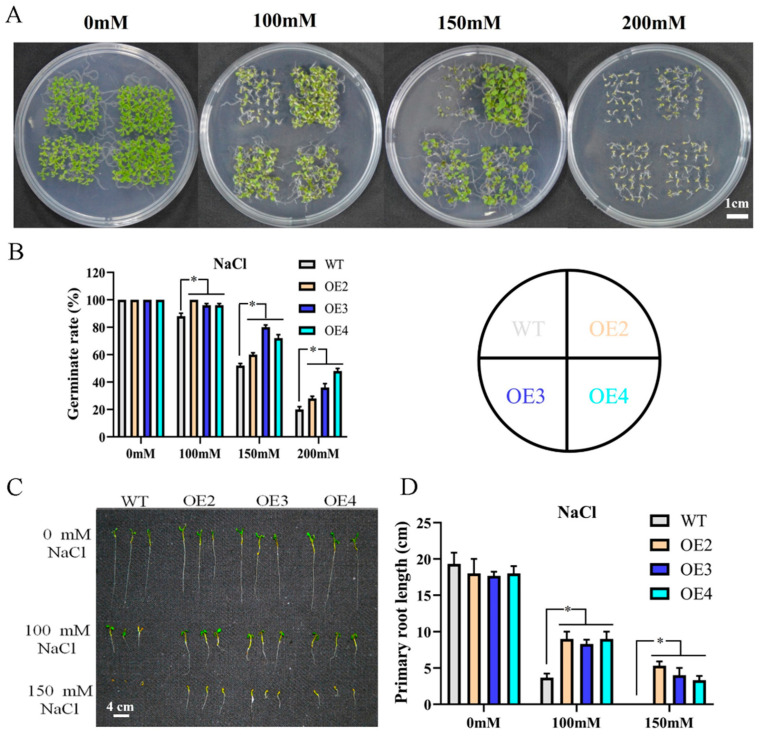
Stress response of transgenic *Arabidopsis* plants expressing *MdMYB108L*. (**A**) Assaying *MdMYB108L*-overexpressing (OE) and wild-type (WT) *Arabidopsis* seed germination in MS medium supplemented with NaCl (0, 100, 150, or 200 mM) at day 10 after being placed in the light. (**B**) Statistical analysis of *Arabidopsis* seed-germination rates under the indicated conditions. (**C**) Assaying *MdMYB108L*- OE and WT *Arabidopsis* primary root lengths in MS medium supplemented with NaCl (0, 100, 150, or 200 mM) at day 10 after being placed in the light. (**D**) Statistical analysis of *Arabidopsis* primary root lengths. The data represent the mean ± SE of three biological replicates. * indicates significant differences (0.01 ≤ * *p* < 0.05).

To further explore the function of *MdMYB108L* in salt stress, we overexpressed the *MdMYB108L* gene in Arabidopsis. Three lines homozygous for a single copy of a transgene (i.e., the OE2, OE3, and OE4 lines) were selected for further analysis. The seeds of wild-type *Arabidopsis* plants and the three transgenic lines obtained were plated on a solid MS medium containing different concentrations of NaCl (0 mM, 100 mM, 150 mM, or 200 mM) and then cultivated. The base was placed in a constant-temperature light incubator for ten days. The results showed that on a medium containing 0 mM NaCl, the seed germination and growth of the transgenic lines did not significantly differ from those of the wild-type line. The results also showed that the germination rate of the wild-type *Arabidopsis thaliana* seeds was 20% in the presence of 200 mM NaCl, whereas those of the OE2, OE3, and OE4 seeds were significantly higher (36%, 28%, and 48%, respectively; Figure 3B). Similarly, under normal conditions, no differences were observed in the taproot length of each line. In a medium containing NaCl, the taproot lengths of all plants were shortened, but the root lengths of the transgenic *Arabidopsis thaliana* plants were significantly higher than those of the control group (Figure 3D). These results indicate that *MdMYB108L* overexpression improves the tolerance of transgenic *Arabidopsis thaliana* lines to salt stress.

### 2.4. Enhanced Salinity Tolerance Possibly Occurred through Reduced ROS Accumulation

In order to further explore the growth status of transgenic and wild-type *Arabidopsis thaliana* under salt stress, we cultured the transgenic and wild-type Arabidopsis for 3 weeks, treated it with 200 mM NaCl aqueous solution for 7 days, and observed the phenotype (Figure S1).

**Figure 4:**
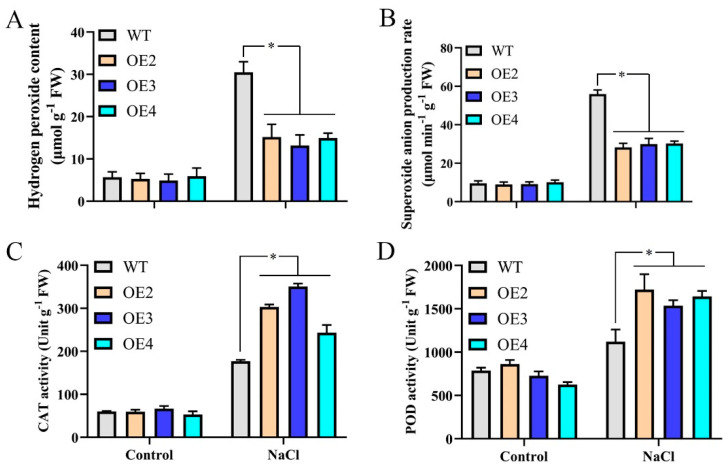
ROS accumulation in *MdMYB108L*-OE *Arabidopsis* seedlings. ROS accumulation and antioxidant enzyme activities of three−week−old *Arabidopsis* seedlings treated with 200 mM NaCl for 7 days. (**A**) Hydrogen peroxide contents. (**B**) Superoxide-anion generation rates. (**C**) CAT activities. (**D**) POD activities. The data represent the mean ± SE of three biological replicates. * indicates significant differences (0.01 ≤ * *p* < 0.05).

When plants are exposed to salt stress, oxidative stress occurs at the subcellular level, triggering ROS production [42]. ROS accumulation causes many adverse effects in plants. To explore ROS accumulation in transgenic *Arabidopsis thaliana* overexpressing MdMYB108L under salt stress, we measured the production rates of hydrogen peroxide and superoxide anions in different plants. The results showed that hydrogen peroxide content was lower in transgenic plants exposed to salt stress. The superoxide-anion content generation rates were significantly lower than those of wild-type *Arabidopsis thaliana* (Figure 4A,B). When plants accumulate high ROS levels, they use antioxidant enzymes to remove excess ROS, enabling resistance to adversity and stress. We, therefore, measured the activities of the four antioxidant enzymes, CAT, and peroxidase (POD). The results showed that after NaCl treatment, compared with wild-type Arabidopsis thaliana, the CAT and POD enzyme activities in transgenic Arabidopsis plants were significantly lower (Figure 4C,D). These findings indicate that under salt stress, the enhanced ability of transgenic Arabidopsis plants to scavenge ROS may be related to increased CAT and POD activities.

### 2.5. NaCl Treatment Improved the Photosynthetic Capacity of Transgenic Apple Hairy Roots

**Figure 5:**
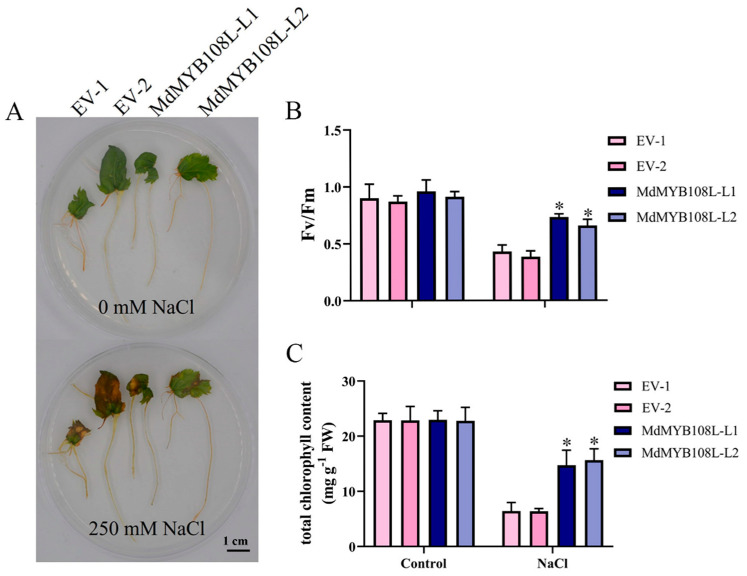
Stress responses of *MdMYB108L*-transgenic apple hairy roots. The transgenic and wild-type lines were cultured on a 1/2 MS medium containing 250 mM NaCl for 15 days. (**A**) Hairy root growth. (**B**) Fv/Fm. (**C**) Total chlorophyll content. The data represent the mean ± SE of three biological replicates. * indicates significant differences (0.01 ≤ * *p* < 0.05). Bar = 1 cm.

The transgenic and wild-type lines were cultured on a 1/2 MS medium containing 250 mM NaCl for 15 days. The results showed that the leaves of the wild-type lines presented obvious yellowing and wilting under salt stress, whereas the leaves of the transgenic lines maintained a firm and emerald-green state (Figure 5A). In addition, adversity stress can negatively impact the photosynthetic capacities in plants. In the absence of salt stress, no significant difference was observed in Fv/Fm between the transgenic and wild-type apple hairy root tissue (leaves). After exposure to salt stress, the PSII primary light-energy conversion efficiency of the transgenic and wild-type hairy root tissue (leaves) significantly decreased, and compared with wild-type apple hairy root tissue (leaves), the PSII original light-energy conversion efficiency of the transgenic lines decreased more moderately (Figure 5B). Similarly, comparable total chlorophyll contents were found between wild-type hairy root tissue (leaves) and transgenic hairy root tissue (leaves) (Figure 5C). These results show that *MdMYB108L* overexpression can help plants withstand salt stress by reducing its impact on the photosynthetic abilities of transgenic plants.

### 2.6. Effects of Injecting an MdMYB108L-Expression Vector on the Expression of Apple-Related Salt Resistance Genes

**Figure 6:**
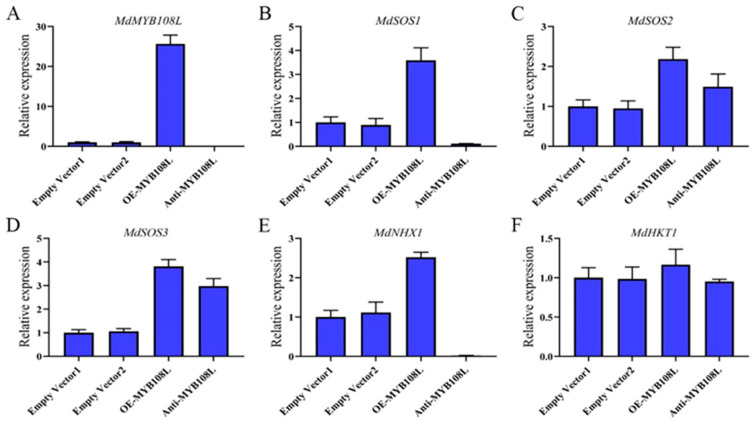
Relative expression levels of different genes in apple peel after transformation with an *MdMYB108L*-overexpression and *MdMYB108L*-silence vector. The apple skin was injected with pGREEN-62-SK, named empty vector1; with TRV-2, named empty vector2; with pGREEN-62-SK-MdMYB108L, named OE-MYB108L, and with TRV2-MdMYB108L, named Anti-MYB108L. (**A**) *MdMYB108L* expression. (**B**) *MdSOS1* expression. (**C**) *MdSOS2* expression. (**D**) *MdSOS3* expression. (**E**) *MdNHX1* expression. (**F**) *MdHKT1* expression.

To determine whether the transcription factor MdMYB108L participates in regulating the expression of salt tolerance genes in apple fruits, we constructed the pGREEN62-SK-*MdMYB108L* overexpression vector and the TRV2-*MdMYB108L* silencing vector, and transformed both vectors into *Agrobacterium* GV3101 cells to prepare for the infection. The infection solution was injected into the epidermal tissue of apple peel, followed by culturing in the dark at room temperature and removal of the peel around the injection site 2 days later. qRT-PCR analysis showed that *MdMYB108L* expression increased significantly in the peel areas surrounding the injection sites (following injection with the overexpression vector), whereas *MdMYB108L* expression significantly decreased in the peel areas in cases where the silencing vector was injected (Figure 6A). Regarding the relative expression levels of salt resistance-related genes, *MdSOS2*, *MdSOS3*, and *MdHKT1* expression levels did not correlate with that of the *MdMYB108L* gene, whereas the *MdSOS1* and *MdNHX1* genes significantly correlated with *MdMYB108L* expression (Figure 6). The transient expression experiments with the apple peel revealed that the transcription factor MdMYB108L might be involved in the transcriptional regulation of *MdSOS1* and *MdNHX1*.

### 2.7. MdMYB108L Directly Upregulated MdNHX1 Expression

**Figure 7:**
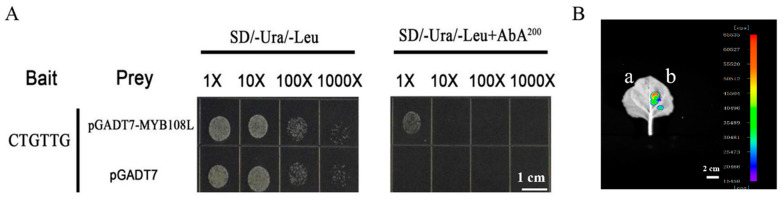
MdMYB108L directly upregulates *MdNHX1* expression. (**A**) Analyses of MdMYB108L binding to the MdNHX1 promoter in yeast-one hybrid assays. (**B**) Analyses of MdMYB108L-dependent regulation of MdNHX1-promoter activity. pGREEN62-SK+MdNHX1pro-LUC ^a^; pGREEN62-SK-MdMYB108L+MdNHX1pro-LUC ^b^.

We analyzed the *MdSOS1* and *MdNHX1* promotors and found that *MdNHX1* had an MYB-binding site. We further studied whether MdMYB108L can bind to the *MdNHX1* promoter and identified the vector pAbAi-*MdNHX1pro* (containing the promoter region). In contrast, untransformed yeast cells could not grow normally (Figure 7A). These results show that the transcription factor MdMYB108L can bind to the promoter of the *MdNHX1* gene. Transcriptional activation activity of the transcription factor MdMYB108L at the *MdNHX1* promoter was detected by performing luciferase assays. The results showed that compared with the no-loading control, the promoter fragments overexpressing *MdMYB108L* and *MdNHX1* had evident transcriptional activation (Figure 7B). We speculate that a binding site exists for the transcription factor MdMYB108L in the promoter of *MdNHX1*. In addition, the transcription factor MdMYB108L positively regulated the transcriptional regulation of *MdNHX1*.

## 3. Discussions

In plants, most MYB proteins belong to the R2R3-MYB subfamily, and their members are of great significance in regulating abiotic stresses in plants. In apples, MdMYB88 and MdMYB124 act as direct regulators of *MdCSP3*, thereby positively regulating the freezing tolerance of apples and the expression of cold stress-response genes [43]. Overexpression of the wheat *TaMYB73* gene in *A. thaliana* enhanced the tolerance of transgenic plants to NaCl and upregulated the expression of *AtCBF3*, *AtABF3*, *AtRD29A*, *AtRD29B*, other salt stress-related genes, and different stress-response genes [44]. In *Arabidopsis thaliana*, AtMYB15 can enhance the resistance of plants to salt and drought [45]. It was also shown that AtMYB20 inhibited the resistance of transgenic plants to drought stress [46]. Heterologous expression of the soybean *GmMYBJ1* gene in Arabidopsis enhanced the tolerance of transgenic *Arabidopsis thaliana* to drought and cold stress [19].

Structural analysis of the MdMYB108L protein showed that the protein has a typical R2R3 domain (Figure 1A) and that its transcriptional activation activity resides in the N-terminal domain (Figure 1B). Preliminary experiments indicated that *MdMYB108L* might play an important role in salt stress (Figure 2A), and MdMYB108L overexpression in yeast might improve tolerance to salt stress (Figure 2B). In addition, under salt stress, the germination rate of transgenic *Arabidopsis thaliana* seeds overexpressing *MdMYB108L* increased significantly, as did the main root length (Figure 3), indicating that MdMYB108L can improve the tolerance of transgenic plants to salt stress.

Adversity stress can cause high ROS accumulation in plants, destroying cell membranes and macromolecular substances, ultimately affecting plant growth and development [47]. Under salt stress, excessive ROS accumulation destroys the structures of key cells in plant tissue through various mechanisms, including membrane lipid peroxidation in the cell membrane, DNA damage, protein denaturation, carbohydrate oxidation, pigment decomposition, and impaired enzymatic activity [48]. The experimental results of this study show that under salt stress, wild-type Arabidopsis plants accumulated more ROS than transgenic *Arabidopsis thaliana* (Figure 4). Healthy plant cells use antioxidant systems to produce and scavenge free radicals, helping maintain homeostasis in normal tissue. However, when plants are under stress conditions, free radicals accumulate. If the protective enzyme system cannot control excessive free radicals, plants can be damaged [49]. Antioxidant enzymes, such as APX, CAT, SOD, and POD, are the main enzymes that remove excess ROS. A transgenic Arabidopsis line that overexpressed the maize *ZmMYB31* gene had higher SOD and APX enzyme activities and lower ROS contents, improving cold resistance [50]. In addition, many transcription factors can regulate the ROS contents in plants through non-enzymatic antioxidant systems, reducing ROS toxicity to plants. In *Arabidopsis thaliana*, overexpression of the wheat *TaNAC2* gene can improve the tolerance of transgenic plants to low temperature, drought, and salt stress by regulating the AsA-GSH cycle [51], and *SlNAC2* overexpression rendered transgenic *A. thaliana* salt tolerant [52]. The results of this study showed that under salt stress treatment, the CAT activities of transgenic *Arabidopsis thaliana* did not differ significantly from those of wild-type plants. The POD activity increased significantly, whereas the APX and SOD activities did not change significantly (Figure 4). These findings show that under salt stress, the enhanced ability of transgenic *Arabidopsis thaliana* to scavenge active oxygen may be related to increased plant antioxidant enzyme activities. In addition, transgenic Arabidopsis plants can attain increased salt tolerance via increased antioxidant enzyme activities.

Photosynthesis is an important process in terms of plant materials and energy conversion, and stress adversely affects the photosynthetic capacities of plants [53]. Salt stress can disrupt photosynthesis, decreasing plant growth and productivity [54]. During salt stress, sodium ion accumulation in cells changes the K^+^:Na^+^ ratio, affecting photosynthesis [55]. In plants, salt stress can also inhibit the light-energy conversion efficiency of PSII [56]. Previous data showed that the chlorophyll contents in salt-tolerant plants are higher than in salt-sensitive plants [57]. In this experimental study, no significant difference occurred between the PSII primary light-energy conversion efficiency of the transgenic and wild-type apple hairy root tissue (leaves) in the absence of salt stress. However, after salt stress treatment, the PSII original light-energy conversion efficiency of the transgenic line was significantly higher (Figure 5B). Wild-type hairy root tissue (leaves) and transgenic hairy root tissue (leaves) showed similar total chlorophyll contents (Figure 5C). These findings indicate that after salt stress, the photosynthetic capacity of *Arabidopsis thaliana* overexpressing MdMYB108L was less affected by salt stress.

Excessive intake of sodium ions may be toxic to plant cells. Generally, plants respond to excess sodium ions through two main strategies: extracellular ion extrusion or intracellular ion isolation. The vacuole Na^+^/H^+^ antiporter (NHX1) and plasma membrane Na^+^/H^+^ antiporter (SOS1) promote the isolation of sodium ions from the cytoplasm to vacuoles, followed by excretion to the extracellular space [30,58]. NHX1 transports Na^+^ to vacuoles by exchanging Na^+^ with the vacuolar H^+^-ATPase (V-ATPase) in vacuoles and the H^+^ pyrophosphatase (V-PPase) in the vacuole membrane via H^+^ electrochemical gradient-dependent exchange [59]. In addition to Na^+^, the upregulation of *AtNHX1* transcription in KCl-treated plants suggests that NHX1 may also help maintain K^+^ homeostasis in cells [60]. To date, eight NHX genes have been identified in the *Arabidopsis thaliana* genome. These are expressed on the membranes of different intracellular organelles and participate in various important functions, such as ion and pH homeostasis, stomatal conductance, protein processing, and cell expansion [61,62]. When plants are exposed to salt stress, genes of the NHX1 family are expressed in *Arabidopsis thaliana* (including AtNHX1, AtNHX2, and AtNHX5) [53], and in *Populus euphratica* PeNHX1, PeNHX2, PeNHX3, PeNHX5, and PeNHX6, among others) [54]. We employed transformation to transiently overexpress or silence MdMYB108L in apple peel and then observed the relative expression of MdNHX1. The results showed that silencing *MdMYB108L* expression in the apple peel nearly abolished MdNHX1 expression in the peel, whereas *MdMYB108L* overexpression in the apple peel caused MdNHX1 to double (Figure 6). The yeast one-hybrid and tobacco transient transcription activation experiments showed that the transcription factor MdMYB108L could bind to the *MdNHX1* promoter, activating *MdNHX1* transcription (Figure 7). These results indicate that the transcription factor MdMYB108L can increase the tolerance of plants to salt stress by transcriptionally regulating MdNHX1 expression.

In conclusion, in this study, we characterized an R2R3-type MYB protein, MdMYB108L, which plays an active role in regulating salt stress. The enhancement of salt tolerance conferred by MdMYB108L overexpression may be achieved by increasing the ability of plants to scavenge ROS. MdMYB108L also positively regulated the expression of the salt tolerance-related apple gene *MdNHX1* to promote plant tolerance to salt stress. Considering that MdNHX1 is related to maintaining the steady state of plant ions, it will be important to determine how MdMYB108L regulates the steady state of plant ions through MdNHX1 in the future.

## 4. Materials and Methods

### 4.1. Plant Materials and Growth Conditions

‘Gala’ apple seedlings were treated with 200 mM NaCl, and samples were obtained at 0, 1, 2, 4, 8, and 12 h post-treatment.

On an ultra-clean workbench, *Arabidopsis* seeds were sterilized by successive treatment with 75% alcohol and 5% sodium hypochlorite, rinsed three times with sterile water, and spread flat on MS medium (containing different concentrations of NaCl), then placed in a constant-temperature light incubator at 23 °C, with a 16 h light/8 h dark cycle. The phenotype was observed after seven days of culture.

After the Arabidopsis seeds were vernalized, they were evenly spread into a flowerpot filled with 3/4 vermiculite and 1/4 nutrient soil. After 3 weeks of normal culture, the seeds were treated with an aqueous solution of 200 mM NaCl for 7 days, and then their phenotypes were observed.

Apple hairy root tissue samples were placed on a clean bench on 1/2 MS medium containing 250 mM NaCl and cultured for 15 days at 25 °C, with a 16 h light/8 h dark cycle. Subsequently, the phenotypes of the samples were observed.

### 4.2. Identification of Transcriptional Activation Activity

An amount of 100 µL Y2H competent yeast cells was thawed on ice and mixed (by pipetting) with 2–5 µg of pre-chilled target plasmid, carrier DNA (10 µL; denatured by performing two cycles of heating at 95–100 °C for 5 min with rapid cooling in an ice bath), and PEG/LiAc (500 µL). The resulting mixture was incubated in a 30 °C water bath for 30 min, transferred to a centrifuge tube and incubated in a 42 °C water bath for 15 min, centrifuged at 5000 rpm for 40 s to discard the supernatant, resuspended in 400 µL di-deoxy H_2_O (ddH_2_O), centrifuged for 30 s to discard the supernatant, and resuspended in 50 µL ddH_2_O. Subsequently, the cells were plated and incubated at 29 °C for 48–96 h. Single colonies with transcriptional activation activity were selected on a chromogenic medium based on the presence of a blue color.

### 4.3. Transforming Yeast Strains and Apple Hairy Root Tissue

We mixed 100 µL of competent yeast cells (*Saccharomyces cerevisiae*) with 1–3 µg of pre-cooled target plasmid, 5 µL of carrier DNA, 500 µL of PEG/LiAc conversion solution, and 20 µL of dimethyl sulfoxide. The resulting mixture was incubated at 42 °C in a water bath for 15 min and centrifuged at 12,000 rpm to discard the supernatant. Next, 1 mL of YPD Plus medium was added to each tube at 30 °C, and the cells were resuspended by shaking at 200 rpm for 1 h. Subsequently, the cells were centrifuged and resuspended in 100 µL 0.9% NaCl, and incubated at 30 °C for 48–96 h.

We screened for *MdMYB108L*-positive Agrobacterium with a configuration solution, then mixed it into an infection solution. Next, leaves growing in a tissue culture flask were cut off, dipped in the infection solution for 2 s, placed on sterile filter paper to absorb the bacteria from the solution, and transferred to a co-culture medium (1/2 MS + 30 g/L sucrose + 7.5 g/L agar, pH 5.2). Then, co-cultivation was carried out in the dark at 22 °C for 3 d. The infected apple hairy roots were cultured at 25 °C in a medium containing antibiotics for 15 days, with a 16 h light/8 h dark cycle.

### 4.4. Active Oxygen Content Determination

Leaves (0.5 g) were ground with 3 mL acetone solution, centrifuged to remove the supernatant, and mixed with 5% titanium tetrachloride solution (0.1 mL) and concentrated ammonia water (0.2 mL). The supernatant was then discarded, and the precipitate was washed with acetone 4–6 times until the color became light, after which 5 mL 2 mol/L H_2_SO_4_ solution was added, and absorbance was measured at 415 nm [63].

Briefly, leaves (0.5 g) were ground and centrifuged, and then the supernatant (0.5 mL) was aspirated. Next, 0.5 mL 50 mmol/L phosphate buffer (pH 7.8) and 1 mL 1 mmol/L hydroxylamine hydrochloride solution were added, and each sample was incubated at 25 °C for 1 h. Subsequently, 1 mL 17 mmol/L p-aminobenzenesulfonic acid and 1 mL 7 mmol/L α-naphthylamine were added, and the temperature was maintained at 25 °C for 20 min. The absorbance was measured at 530 nm [63].

### 4.5. Antioxidant Enzyme Activity Assays

Leaves (0.5 g) were ground and centrifuged at 12,000× *g* for 20 min at 4 °C, and the antioxidant enzyme activity in the crude supernatant was determined.

Determination of CAT activity: 0.28 mL of 30% H_2_O_2_ was diluted to 250 mL with pH7.0 phosphate buffer, which is the CAT reaction solution. Amounts of 2.9 mL of CAT reaction solution and 0.1 mL of crude enzyme extract were pipetted and quickly mixed. The absorbance was measured at 240 nm (10–20 s).

Determination of POD activity: 0.2553 mL of 30% H_2_O_2_ and 0.125 mL of guaiacol were adjusted to 250 mL with pH7.0 phosphate buffer, which was the POD reaction solution. Amounts of 3 mL POD reaction solution and 20 μL crude enzyme extract solution were pipetted and quickly mixed. The absorbance was measured at 470 nm (0–30 s) [64].

### 4.6. Determination of Chlorophyll Fluorescence Parameters and the Total Chlorophyll Content

Chlorophyll fluorescence parameters were determined by first clamping tested leaves with dark-adaptation clips and allowing them to fully adapt to the dark for 15–30 min. Then, the optical measurement window of a portable FMS-2 fluorometer was placed on the dark-adapted leaf clamp. Next, the light-shielding sheet of the clamp was removed, and the maximum photochemical quantum yield (Fv/Fm) of photosynthetic system II (PSII) was measured.

The total chlorophyll content was determined by placing 0.1 g leaves in 15 mL of 96% ethanol, and extracting for 48 h in the dark. After extraction, the absorbance of the sample was measured at 665 nm and 649 nm. The following formula was used for the calculation: total chlorophyll content (mg·g^−1^) = 6.63OD_665_ + 18.08OD_649_ [65].

### 4.7. Luciferase Experiments

The MdNHX1pro-LUC and MdMYB108L-pGREEN62-SK vectors were constructed and used to transform competent *Agrobacterium* GV3101 cells. Briefly, cells were first mixed with the vectors in a 5 mL YEP liquid medium and incubated at 28 °C for 12–16 h. Then, 2 mL of each culture was inoculated into a 50 mL YEP liquid medium, incubated at 28 °C for 4–5 h, and centrifuged at 6000 rpm for 10 min to collect bacteria. Subsequently, bacteria were suspended in a buffer (pH 5.7) containing 10 mM MES, 10 mM MgCl_2_, 150 mM acetosyringone, and ddH_2_O. After resuspension, the collected bacteria were allowed to stand at room temperature for 1–3 h and mixed with a bacterial solution containing the MdNHX1pro-LUC and MdMYB108L-pGREEN62-SK vectors and the promoter at a 1:1 ratio, using a needleless syringe. The bacterial solution was then injected into the tobacco leaf; 2 days after the injection, fluorescein was injected into the same part of the tobacco leaf, and the fluorescence intensity of the leaf was observed with a real-time imager.

### 4.8. Yeast One-Hybrid Experiments

After constructing the MdNHX1pro-pAbAi and MdMYB108L-pGADT7 vectors, the MdNHX1pro-pAbAi vector was digested with Bpil. The digested plasmid was recovered from an agarose gel and used to transform Y1H Gold competent cells. Subsequently, the strain was spread on synthetic dextrose minimal medium without uracil (SD-Ura) and cultivated 2–3 days to select single colonies; correct colonies were used to prepare a mother liquor. Bacteria were then spotted onto a medium containing different concentrations of SD-Ura+Aureobasidin A (AbA) to select an appropriate concentration of AbA and prepare competent yeast cells. The MdMYB108L-pGADT7 vector was transferred into freshly prepared competent yeast cells, the bacterial liquid was coated on SD-Ura-Leu medium, and single bacterial colonies were grown for screening and identification purposes. Correct colonies were transferred into the mother liquid and diluted, and the bacterial liquid spot on the SD-Ura-Leu+AbA medium (containing the AbA concentration determined as described above) was assessed to confirm the yeast one-hybrid results.

### 4.9. Quantitative Real-Time Polymerase Chain Reaction (qRT-PCR) Analysis

The NCBI website was used for primer design; the sequences of the qRT-PCR primer set are shown in Table S1. Kangwei Century UltraSYBR Mixture (Low Rox) reagent was used for the qRT-PCR experiments, which were repeated three times. The thermocycling procedure was as follows: pre-denaturation at 95 °C for 10 min, followed by 40 cycles of denaturation at 95 °C for 15 s, and annealing/extension at 60 °C for 1 min. Three independent biological replicates were assayed for each sample.

### 4.10. Statistical Analysis

Three biological replicates were used for all measurements. The data were averaged over multiple experiments and subjected to analysis of variance (ANOVA) and Duncan’s test using SPSS software (version 22.0). Differences between means were considered statistically significant at *p* < 0.05.

## Data Availability

Not applicable.

## Supplementary Materials

The following supporting information can be downloaded at: https://www.mdpi.com/article/10.3390/ijms23169428/s1.
