# Supplementary material for: Over-Expression of an R2R3 MYB Gene, MdMYB108L, Enhances Tolerance to Salt Stress in Transgenic Plants
Source: Int J Mol Sci. 2022 Aug 21;23(16):9428. doi: 10.3390/ijms23169428 (PMC9409463; doi:10.3390/ijms23169428)
Supplement: Supplementary file 1 [file ijms-23-09428-s001.zip › ijms-1826607-supplementary/Supplemental files/Supplemental Figures.pdf]

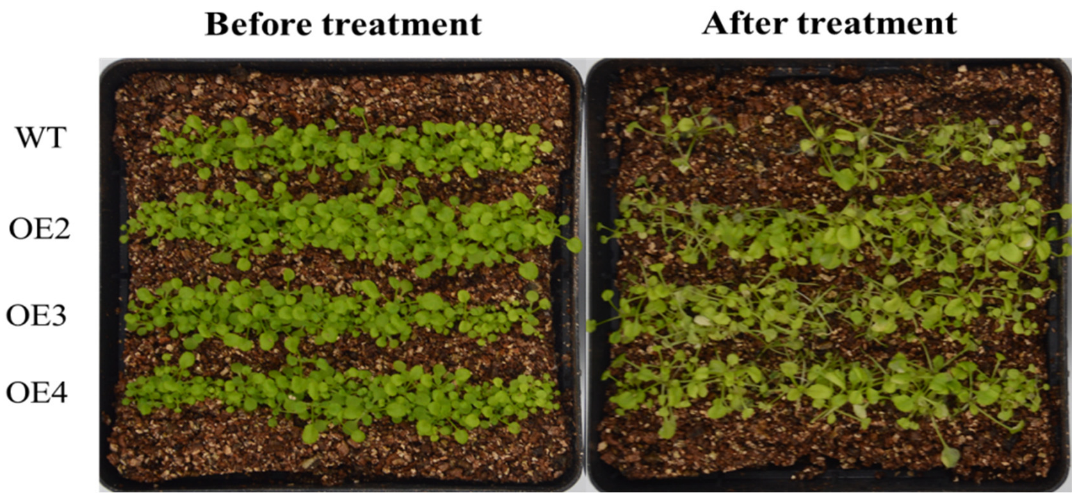

Supplemental Figure S1. The phenotypes of WT and transgenic *Arabidopsis* plants under salt stress. The transgenic and wild type *Arabidopsis* for 3 weeks, treated it with 200 mM NaCl aqueous solution for 7 days.
