# Supplementary material for: Over-Expression of an R2R3 MYB Gene, MdMYB108L, Enhances Tolerance to Salt Stress in Transgenic Plants
Source: Int J Mol Sci. 2022 Aug 21;23(16):9428. doi: 10.3390/ijms23169428 (PMC9409463; doi:10.3390/ijms23169428)
Supplement: Supplementary file 1 [file ijms-23-09428-s001.zip › ijms-1826607-supplementary/Supplemental files/Supplemental Table.pdf]

Table S1 Primers for gene amplification and qRT-PCR

Primer	Forward Primer sequence (5′-3′)	Reverse Primer sequence (5′-3′)
MdMYB108L	ATGGATATTAATAATGTTCATGATCA	GTCATCACTAAGCTGTTGTTGC
MdMYB108L-C	ATGGATATTAATAATGTTCATGATC	GTTAACGTCACATTTGAGT
MdMYB108L-N	AGCAAACAATTTCAAG	GTCATCACTAAGCTGTT
MdNHX1pro	GACATCAACGTGGATGAAGG	CCACCTCTTGGAATAAATTACAC
MdActin	GGCTGGATTTGCTGGTGATG	TGCTCACTATGCCGTGCTCA
qMdMYB108L	TGAGATCAGCACGTTATGGC	ATTTGGTCGCAAGTAGTTTA
qMdSOS1	AGGAAACCATGAAATTGTGTGG	GATCATGTCACAAATGTAGGGC
qMdSOS2	AATCAATGGGTCTCAAGGTC	CCTCCTTCGGTTTCCAAATA
qMdSOS3	GGGGTTATTGAGTTTGGAGA	GGATGCTTCGACACAAATTC
qMdNHX1	AAGCGACAGTCCTGGAACAT	TATTATCACTTGCTGCCGGAG
qMdHKT1	TCGTTCGCTATTTCGTGTCCT	TGGGCCTGAAAGAAGTGTTTG
